# CRISPR/Cas‐based precision genome editing via microhomology‐mediated end joining

**DOI:** 10.1111/pbi.13490

**Published:** 2020-11-09

**Authors:** Tien Van Vu, Duong Thi Hai Doan, Jihae Kim, Yeon Woo Sung, Mil Thi Tran, Young Jong Song, Swati Das, Jae‐Yean Kim

**Affiliations:** ^1^ Division of Applied Life Science (BK21 Plus Program) Plant Molecular Biology and Biotechnology Research Center Gyeongsang National University Jinju 660‐701 Republic of Korea; ^2^ National Key Laboratory for Plant Cell Biotechnology Agricultural Genetics Institute Km 02, Pham Van Dong Road Co Nhue 1, Bac Tu Liem, Hanoi 11917 Vietnam; ^3^ Division of Life Science Gyeongsang National University 501 Jinju‐daero Jinju 52828 Republic of Korea

**Keywords:** MMEJ, precision gene editing, microhomology, CRISPR, cas, DNA repair, PITCh

## Abstract

Gene editing and/or allele introgression with absolute precision and control appear to be the ultimate goals of genetic engineering. Precision genome editing in plants has been developed through various approaches, including oligonucleotide‐directed mutagenesis (ODM), base editing, prime editing and especially homologous recombination (HR)‐based gene targeting. With the advent of CRISPR/Cas for the targeted generation of DNA breaks (single‐stranded breaks (SSBs) or double‐stranded breaks (DSBs)), a substantial advancement in HR‐mediated precise editing frequencies has been achieved. Nonetheless, further research needs to be performed for commercially viable applications of precise genome editing; hence, an alternative innovative method for genome editing may be required. Within this scope, we summarize recent progress regarding precision genome editing mediated by microhomology‐mediated end joining (MMEJ) and discuss their potential applications in crop improvement.

## Background

In nature, DNA breaks occur frequently inside a cell due to endogenous as well as exogenous stimuli. The broken genome should be repaired to maintain its stability; otherwise, it may lead to cellular malfunctioning. Even single double‐stranded break (DSB) damage in chromosomes could lead to cell death if it is not properly repaired (Karanjawala *et al*., 2002; Lindahl, 1993). The commonly held view of DSB repairs is that the direct ligation of the broken ends by non‐homologous end joining (NHEJ) without leaving any ‘DNA scars’ such as insertion or deletion of a few base pairs is an ideal scenario for cells. Cells have evolved DSB repair machinery that is strong enough to maintain chromosome integrity for their survival. However, the extensive introduction of DSBs by non‐biological (gamma radiation or DSB‐inducing chemicals) or biological (site‐directed nucleases (SDNs)) agents lead to error‐prone repairs with DNA scars (Vu *et al*., 2019).

Molecular precision editing has become a common approach since the advent of site‐directed nucleases (SDNs), including rapidly emerging clustered regularly interspaced short palindromic repeats (CRISPR)/CRISPR‐associated (Cas) protein complexes (Barrangou *et al*., 2007; Jansen *et al*., 2002). The power of CRISPR‐based precision gene editing comes from the ability to introduce DSBs into theoretically any specific sites (genes) in a genome of interest (Jinek *et al*., 2012). The majority of SDN‐based gene editing approaches take advantage of the erroneous but highly efficient NHEJ DSB repair processes to successfully generate edited but seemingly unpredictable events. The consequences of DSB damage are widely varied, from single base changes to chromosome‐scaled modifications (Puchta, 2005) that lead to changes in the genotype of the targeted organism. DSBs could also be precisely repaired by the homologous recombination (HR) pathway if donor DNAs with homologous ends are present at the broken hot spot and under favourable cellular conditions, but this occurs at extremely low frequency in plants. The latter approach has been conferred as the major way to precisely repair DSBs and offers great potential for precision crop improvement. Using positive–negative selection (Nishizawa‐Yokoi *et al*., 2015; Terada *et al*., 2002), SDN‐based DSB formations (Li *et al*., 2016; Qi *et al*., 2013; Sun *et al*., 2016; Wright *et al*., 2005) and DNA replicon‐based delivery of donor templates (Cermak *et al*., 2015; Gil‐Humanes *et al*., 2017; Wang *et al*., 2017), HR‐based gene targeting (HGT) in plants was extensively studied to improve the frequency of this approach. However, it is still challenging to attempt to edit an allele by HGT without using any allele‐associated selection marker.

Recently, to expand the scope of precision gene targeting in animals and human cells, several studies have been successfully conducted to engineer the NHEJ pathway for HR‐independent precision gene editing. One of the approaches is CRISPR/Cas9‐mediated microhomology (MH)‐dependent targeted integration (MITI) or Precise Integration into Target Chromosome (PITCh), an application of MH‐mediated end joining (MMEJ) repair of DNA DSBs (Ata *et al*., 2018; Hayashi and Tanaka, 2019; Li *et al*., 2019; Nakade *et al*., 2014; Sakuma *et al*., 2016; Shin *et al*., 2018; Yao *et al*., 2017b). Considering the dominance and high efficiency of NHEJ in all of the cell cycles and types, the approach may pave novel ways for precision crop improvement. In this review, we discuss the molecular mechanism underlying the MITI/PITCh approach and suggest some technical opinions for further improvement of the frequency of this precision gene editing application in plants.

## MMEJ‐mediated DSB repair mechanism

**Figure 1:**
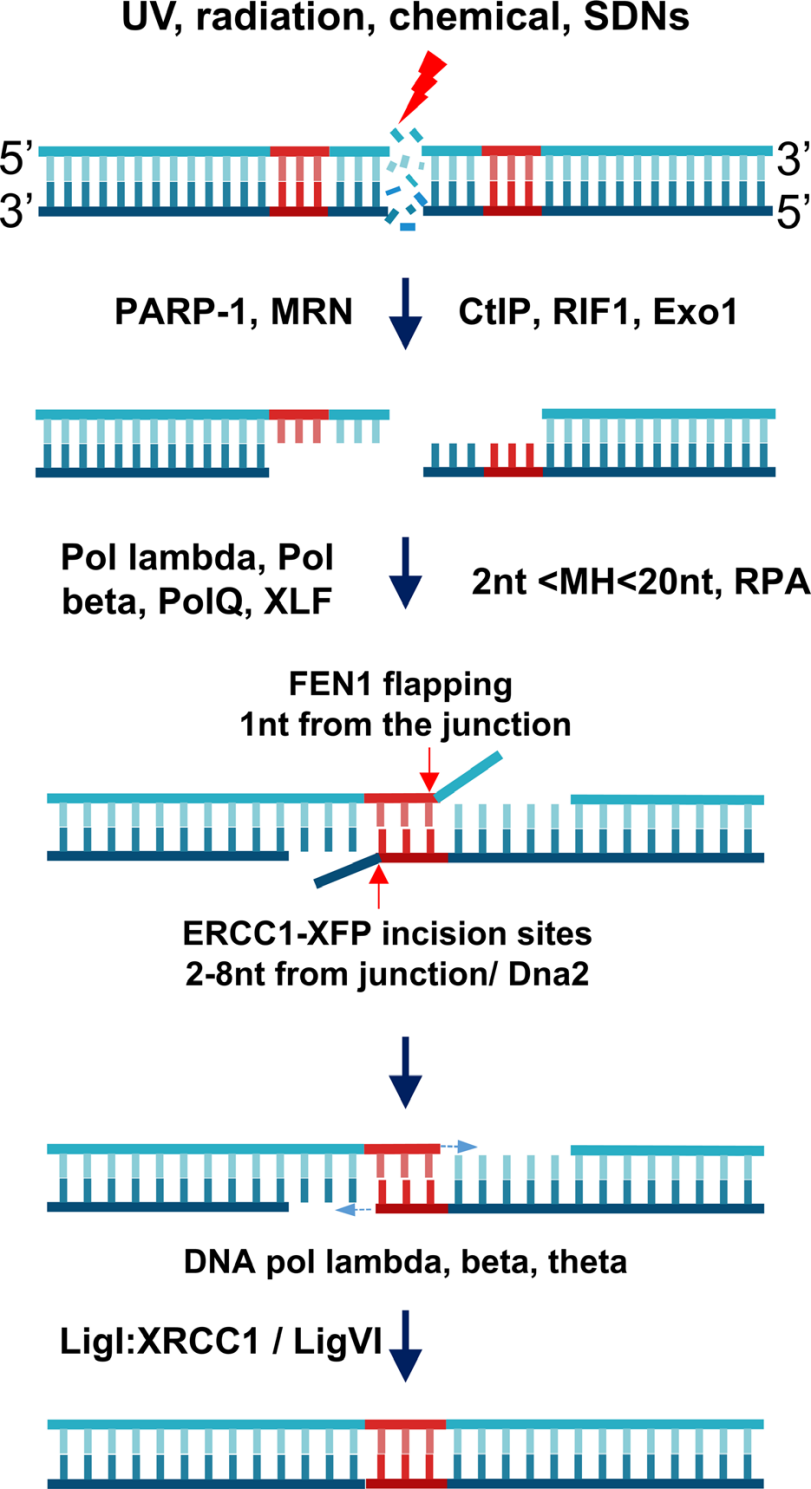
MMEJ‐mediated DSB repair. DSBs formed by environmental stimuli or artificial SDNs are sensed by DSB sensors for MMEJ, such as poly(ADP‐ribose) polymerase‐1 (PARP‐1), which subsequently activate and recruit DNA damage repair proteins, such as the end resection complex MRN and CtIP, with supportive roles from RIF1 and exonuclease 1. End resection results in 3′ overhangs that, if containing MHs (preferred lengths approx. 2–20 nucleotides), activate MMEJ. The 3′ overhangs with MHs prime annealing of the MHs and their 3′ flaps are incised by ERCC1‐XFP or flapped by flap endonuclease 1 (FEN1) (which does not have strong activity for 3′ flaps). The 3′OH processed ends could further prime DNA‐dependent DNA polymerization to fill the gap between the annealed MHs and the other 5′ ends. Filled gaps appear as nicked sites that, if the 5′ termini have phosphate groups, are directly ligated by ligase I complex or DNA ligase VI to a lesser extent. If the 5′ ends do not have free phosphate groups, the nicked sites trigger base excision or nucleotide excision repair mechanisms to correct the sites. Red arrows: junctions. [Colour figure can be viewed at wileyonlinelibrary.com]

MH‐dependent repairs were first traced from *E. coli* populations with deletions of sequence mediated by linearized DNA repair, viral genome insertions in mammals (Roth *et al*., 1985; Ruley and Fried, 1983), T‐DNA integration in plants (Gheysen *et al*., 1991; Mayerhofer *et al*., 1991) and DSB repairs in budding yeast (Kramer *et al*., 1994). Typical characteristics of this type of repair were multiple recombination events occurred within a limited area (~50 bp) of DNA at the non‐homologous DNA’s linearized ends, which contained short stretch(s) of sequence homology (2–20 bp). The microhomologous sizes may reveal distinct repair machinery requirements (Villarreal *et al*., 2012). Repaired products frequently contain sequence deletions limited to the nucleotides located between the microhomologous sequences. Furthermore, the frequencies of the MH‐directed repairs were shown to be as high as that of the canonical non‐homologous end‐joining (cNHEJ) mechanism (Roth and Wilson, 1986; Tan *et al*., 2020). The repair outcomes were later revealed to be different from those of cNHEJ through studies using cNHEJ‐deficient yeast cells (Boulton and Jackson, 1996), calf thymus fractionated extracts (Mason *et al*., 1996) and plants (Tan *et al*., 2020; Weiss *et al*., 2020). The novel DNA DSB repair mechanism, an alternative to cNHEJ, was referred to as microhomology‐mediated end joining (MMEJ) (Klugbauer *et al*., 2001; Figure 1) or MHEJ (Zhong *et al*., 2002).

**Figure 2:**
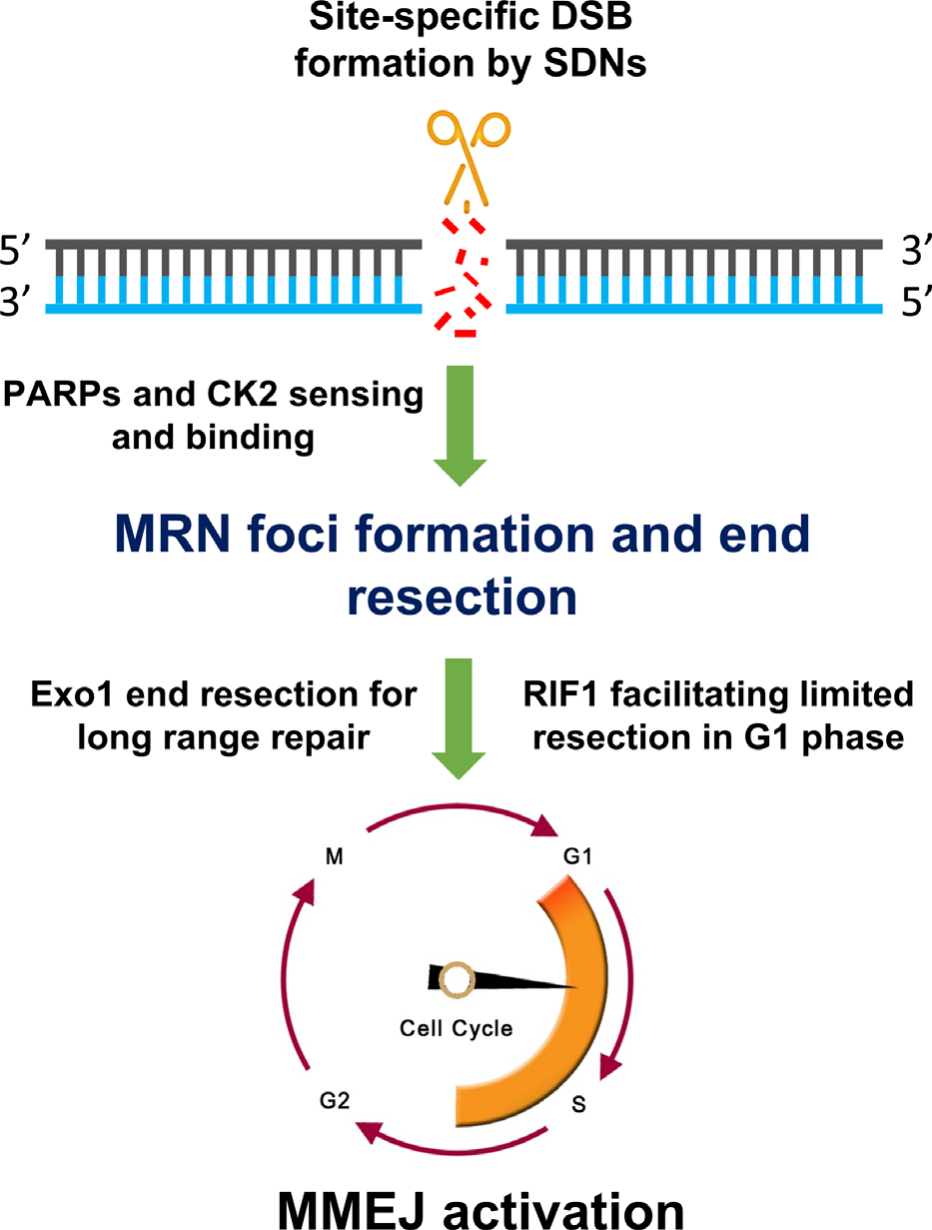
MMEJ activation. PARP‐1 and CK2 work in coordination as DSB sensors to bind to broken ends and activate themselves and other DSB repair proteins to generate MRN end resection foci, thereby unmasking MHs and activating MMEJ. Exo1 and RIF1 play supplemental roles in end resection in some conditions. [Colour figure can be viewed at wileyonlinelibrary.com]

MMEJ was considered to be a back‐up DSB repair mechanism when it was first observed; however, in recent reports, it was shown to be another major DSB repair pathway in yeast and mammals (Ata *et al*., 2018; McVey and Lee, 2008; Sfeir and Symington, 2015; Sharma *et al*., 2015), and it was found to be active during G1 and early S phases (Figure 2; Taleei and Nikjoo, 2013). The mechanism of MMEJ has been proposed to include at least five steps: (i) DSB end resection; (ii) annealing of MHs; (iii) removal of 3′ flaps; (iv) filling in the gap remaining after the removal of the ends; and (v) sealing the nicks, which are discussed in the following sections.

### DSB end resection

The components of the DSB end resection machinery of MMEJ are largely shared with those of HR (Sfeir and Symington, 2015). A limited range of MHs become unmasked by 5′‐3′ end resection of DSB ends by MRX/MRN to form short 3′ overhangs containing microhomologies (Ma *et al*., 2003; Sharma *et al*., 2015; Taylor *et al*., 2010; Truong *et al*., 2013). Initially, in the absence/competition of KU heterodimers, poly(ADP‐ribose) polymerase‐1 (PARP‐1) (Table 1) might detect and then occupy DSB ends (Wang *et al*., 2006) and activate itself by adding poly(ADP‐ribose) groups to lysine residues in the activation domain (AD) (Altmeyer *et al*., 2009), which occurs in a transient manner (Thomas *et al*., 2019). Activated PARP‐1 might subsequently target glutamate residues of histone H1 proteins to relax the chromatin and activate other DNA repair proteins or sustain their activities (Muthurajan *et al*., 2014; Thomas *et al*., 2019). PARP‐1 was shown to be important for the assembly or stability of X‐ray repair cross‐complementing 1 (XRCC1) foci in response to oxidative DNA damage (El‐Khamisy *et al*., 2003). XRCC1 phosphorylation by casein kinase 2 (CK2) might enhance its interaction with MRE11 and CtIP, which were shown to be involved in DSB end resections and thereby activate the MMEJ repair pathway (Figure 2; Dutta *et al*., 2017; Yun and Hiom, 2009). In the absence of other 5′‐3′ end resection enzymes, the 5′‐3′ exonuclease Exo1 might also be involved in DSB end processing for MMEJ (Decottignies, 2007; Tomita *et al*., 2003) at long‐range microhomologies (Sfeir and Symington, 2015). On the other side, the resection could be suppressed by cNHEJ‐related components such as ATM (Rahal *et al*., 2010) and LIG4 (Simsek *et al*., 2011).

**Table 1:** The major MMEJ‐associated proteins among yeast, animal and *Arabidopsis* summarized in this review

Yeast	Animal	*Arabidopsis*	Identity to the human ortholog (%)^†^	MMEJ‐related biochemical functions	MMEJ step(s)	References (plant‐specific)
N/A	PARP1	AtPARP1 (AT2G31320)	38.09	Poly(ADP‐ribosyl)ation	Activation	Doucet‐Chabeaud et al. (2001)
SIR2	SIRT6	N/A	–	Poly(ADP‐ribosyl)ation	Activation	Tanny et al. (1999)
MRE11	MRE11	AtMRE11 (AT5G54260)	39.52	3′ to 5′ Exonuclease and Endonuclease Activities	DSB End Resection	Hartung and Puchta (1999)
RAD50	RAD50	AtRAD50 (AT2G31970)	30.79	ATPase Activity	DSB End Resection	Gallego and White (2001)
XRS2	NBS1	AtNbs1 (AT3G02680)	26.02	Nuclear localization and regulation of the catalytic activities of both Mre11 and Rad50	DSB End Resection	Akutsu et al. (2007); Waterworth et al. (2007)
SAE2	CtIP	AtGR1/AtCOM1 (AT3G52115)	47.5	3′ to 5′ Exonuclease Activities; Co‐factor of MRN	DSB End Resection	Uanschou et al. (2007)
EXO1	EXO1	AtEXO1A (AT1G29630) and AtEXO1B (AT1G18090)	35.78 and 37.99	5′‐3′ Exonuclease activities	DSB End Resection	Furukawa et al. (2008); Kazda et al. (2012)
RPA70	RPA1	AtRPA70a (AT2G06510); AtRPA70b (At5g08020); AtRPA70c (AT5G45400); and AtRPA70d (AT5G61000)	35.62; 35.58; 39.73; and 32.68	DNA binding activity	MH Annealing	Ishibashi et al. (2005); Takashi et al. (2009)
RPA32	RPA2	AtRPA32a (AT2G24490) and AtRPA32b (AT3G02920)	28.88 and 33.99	DNA binding activity	MH Annealing	Takashi et al. (2009)
RPA14	RPA3	AtRPA14A (AT3G52630) and AtRPA14B (AT4G18590)	22 and 21	DNA binding activity	MH Annealing	Takashi et al. (2009); Aklilu et al. (2014)
RAD52	RAD52	AtRAD52‐1 (AT1G71310) and AtRAD52‐2 (AT5G47870)	13 and 11	Single‐stranded DNA binding activity	MH Annealing	Samach et al. (2011)
RAD27	FEN1	AtFEN1 (AT5G26680)	54.69	Single‐stranded DNA endonuclease activity	Removal of 3′ flaps	Kimura et al. (2000); Zhang et al. (2016)
RAD10	ERCC1	AtERCC1 (AT3G05210)	48.33	Structure‐specific endonuclease activity	Removal of 3′ flaps	Hefner et al. (2003); Dubest et al. (2004)
RAD1	XFP	AtRAD1 (AT5G41150)	39.64	Structure‐specific endonuclease activity	Removal of 3′ flaps	Fidantsef et al. (2000); Gallego et al. (2000)
DNA pol IV	Polλ	AtPolλ (AT1G10520)	37.29	DNA polymerase activity	Filling‐in the gap from the flapped 3′OH	Roy et al. (2011)
PolQ	PolQ	AtTEB (AT4G32700)	40.59	DNA polymerase activity	Filling‐in the gap from the flapped 3′OH	Inagaki et al. (2006)
N/A	LIG3	N/A	–	–	–	–
CDC9	LIG1	AtLIG1 (AT1G08130)	50.32	DNA ligase activity	Sealing the nicks	Taylor et al. (1998); Sunderland et al. (2006)

N/A, Not yet identified.

^†^ Identity revealed by searching Model Organisms (landmark) database using PSI‐BLAST (Position‐Specific Iterated BLAST) (https://blast.ncbi.nlm.nih.gov/Blast.cgi).

### Annealing of MHs

MH lengths may determine the repair pathway among MMEJ, single‐strand annealing (SSA), and HR (Villarreal *et al*., 2012). The 3′ overhangs resulting from 5′‐3′ end resection might be protected by RPA (RPA70, RPA32, and RPA14) (Table 1) heterotrimers to interfere with early spontaneous annealing and repair by MMEJ while supporting SSA or HR (Ahrabi *et al*., 2016; Deng *et al*., 2014). The lengths of microhomologous sequences might be as low as 1 nt (Koole *et al*., 2014), but they have been shown to be the most efficient at 5 nt (Ata *et al*., 2018; Sharma *et al*., 2015). Moreover, high‐throughput analysis of DSB repair mediated by TALENs and CRISPR/Cas‐based molecular scissors in human cells revealed that the major MH range was 2–8 nt (Bae *et al*., 2014). RAD52 was shown to be required for RPA displacement and ssDNA strand annealing during SSA (Symington *et al*., 2014). The spontaneous annealing for MMEJ in mammals might be RAD52‐independent, whereas, in budding yeast, it may require RAD59, a RAD52 homolog, which might be due to longer MHs (Lee and Lee, 2007; Sugawara *et al*., 2000).

### Removal of 3′ flaps

The removal of 3′ flaps (Figure 1) may be mediated by the incision activities of ERCC1‐XPF (Table 1), a structure‐specific heterodimeric endonuclease ortholog of the Rad1‐Rad10 complex in *Saccharomyces cerevisiae* that primarily functions in the nucleotide excision repair pathway (Ahmad *et al*., 2008; Fishman‐Lobell and Haber, 1992; de Laat *et al*., 1998; Tsodikov *et al*., 2005). Specifically, under in vitro conditions, the 3′ flaps lengths required for removal by ERCC1‐XPF were a minimum of 4–8 nucleotides, and incisions occurred at the 5′ side of a junction, at a distance of 2–8 bases from the junction (Figure 1; de Laat *et al*., 1998). This means that the flapped substrates should logically carry more than 2–8 MH‐annealed duplexes. A potential molecule that might be involved in removing the 3′ flaps is flap endonuclease 1 (FEN1) (Table 1), which was first characterized as mature Okazaki fragments during DNA replication and long‐patch base excision repair by 5′ flap removal activities at 1 base from its junction (Gottlich *et al*., 1998; Harrington and Lieber, 1994; Mengwasser *et al*., 2019; Sharma *et al*., 2015; Turchi *et al*., 1994). However, the 3′ flapping activities of mammalian FEN1 might be very low (Harrington and Lieber, 1994), and the activities are even abolished in the case of RPA‐bound ssDNA flaps. The activities of FEN1 were restored with the presence of Dna2, a DNA helicase/nuclease that could displace RPA complexes from DNA flaps and shorten them (Bae *et al*., 2001). Other 3′‐5′ exonucleases, such as EXO1, or high fidelity DNA polymerases, such as DNA pol δ with 3′‐5′ proof‐reading activity, may also be involved in completely removing the remaining 1–2 nt flaps in some products of ERCC1‐XPF or Dna2/FEN1 (Harrington and Lieber, 1994). The flapping process generates free 3′‐hydroxyls that could prime DNA filling‐in by DNA polymerase (Sfeir and Symington, 2015).

### Filling‐in the gap from the flapped 3′OH

The gaps available between the annealed microhomologies and ssDNA‐dsDNA junctions (Figure 1) must be filled in by DNA polymerase prior to the final ligation. DNA pol λ (Table 1), the only member of the Pol X family, appears to be conserved in nearly all kingdoms, including Plantae, and it has also been shown to be involved in MMEJ (Capp *et al*., 2006; Crespan *et al*., 2012). The activities of DNA pol λ were shown to be enhanced by the Rad9/Hus1/Rad1 (9‐1‐1) complex, which might counteract strand blocking by RPA trimers (Crespan *et al*., 2012). Alternatively, DNA polymerase Pol θ, a member of Family A DNA polymerases, could bypass the mismatches and directly extend the imperfect 3′ flapped products (Kent *et al*., 2015; Newman *et al*., 2015). Pol θ was also shown to play a role in blocking by binding to RAD51, thereby supporting MMEJ (Ceccaldi *et al*., 2015).

### Sealing the nicks

The nicked sites formed after the gap‐filling synthesis process should be ligated by a DNA ligase to finish MMEJ‐mediated DNA repair (Figure 1, Table 1). DNA ligase III is involved in the ligation of nicks in BER/NER as well as MMEJ (Gottlich *et al*., 1998; Simsek *et al*., 2011; Wang *et al*., 2005). The ligation activities might be performed through the cooperation between DNA ligase I and DNA ligase III (Liang *et al*., 2008). The major form of DNA ligase III is recruited to the repair foci by direct interaction with XRCC1 via their BRCT domains, whereas DNA ligase I accumulates at the repair sites by PCNA (Mortusewicz *et al*., 2006; Nash *et al*., 1997). However, DNA ligase III, but not DNA ligase I, was shown to be limited to vertebrates, indicating that the last step of the MMEJ pathway may be evolutionally modified in plants. Therefore, DNA ligase I may play important roles in ssDNA break and DSB repair in plants (Waterworth *et al*., 2009). The ligation step of MMEJ in *Arabidopsis* may occur through the association of pol λ, DNA ligase I and, to a lesser extent, DNA ligase VI (Furukawa *et al*., 2015). Finally, DNA ligases were proposed to play roles in the displacement of PARP‐1 from the broken sites through their zinc finger motifs (Mackey *et al*., 1999), which completes the repair procedure.

## Engineering MMEJ for precision gene editing

### Current status

Initially, identified as a backup pathway of DSB repair, MMEJ (Figure 1) has recently been recognized as one of the major DSB repair pathways in addition to cNHEJ and HR (Deriano and Roth, 2013; Sfeir and Symington, 2015; Wang *et al*., 2003). It is widely known that MMEJ frequently leads to DNA deletion and/or rearrangement. MHs were dominant at 43.7% and 39.6% frequencies among all the mutations mediated by TALENs and RNA‐guided endonucleases (RGENs), respectively, and could be precisely predicted (Bae *et al*., 2014).

**Figure 3:**
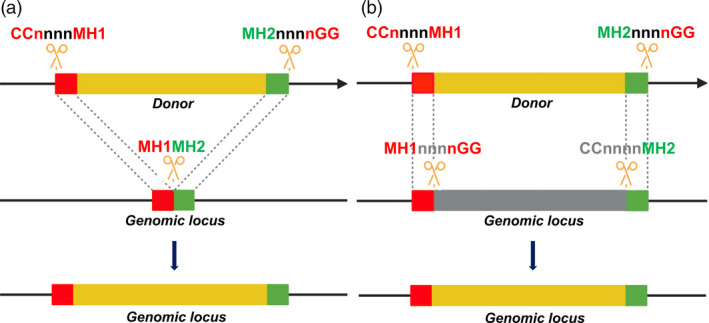
Strategic schemes for donors and genomic loci for MMEJ‐mediated precision editing using CRISPR/SpCas9. (a) MMEJ‐mediated targeted insertion model. (b) MMEJ‐mediated targeted replacement model. MH1 and MH2 are predefined from genomic loci and are subsequently introduced into the two ends of the donor sequence. CRISPR/Cas complexes are designed with canonical PAM binding sites (nGG/CCn) to produce DSBs at donors and genomic loci with MH1 and MH2 flanking ends. MMEJ‐mediated repair using the donor sequence generates genomic loci with precise modifications. [Colour figure can be viewed at wileyonlinelibrary.com]

In the same year, a system called PITCh (Precise Integration into Target Chromosome) that used TALENs and CRISPR/Cas9 for targeted genome modifications via the MMEJ pathway (8‐nt MH) in various animals was successfully engineered (Nakade *et al*., 2014). The important point in designing PITCh was to avoid recurrent cutting by TALENs or CRISPR/Cas complexes. The precise PITCh frequencies in mammalian cells, silkworms (*Bombyx mori*) and frogs (*Xenopus laevis*) were much higher than they were in the HR pathway. Due to the dimeric active forms of FokI, a pair of TALENs must be designed for each of the dsDNA cut sites on the donors and targeted genomic sites. Moreover, to avoid recurrent cutting by the same TALENs after ligation, the junction sites have to be shortened from their original forms, thereby limiting the ability of TALEN‐based PITCh to perform a targeted insertion with unaltered sequences at the junctions. However, the CRISPR/Cas‐based PITCh does not have the latter limitation, as it could be designed to precisely edit genomic loci without resulting in any undesirable sequence modification (Figure 3; Nakade *et al*., 2014). The CRISPR/Cas‐based PITCh system was later advanced using 20‐nt MH at the distal site of DSBs that showed higher precision editing efficiencies (Sakuma *et al*., 2016).

Precise targeting for in vivo, ex vivo and in situ knock‐ins is very important for gene therapy studies. Using similar CRISPR‐based MMEJ‐mediated gene editing approaches, Yao and co‐workers showed efficient MMEJ‐based targeted knock‐in in mouse hepatocytes. With 28‐nt left and 20‐nt right as the MH flanking donor sequences, the knock‐in frequencies were as high as 20%, which was approximately 10‐fold higher than the frequency of the HR‐based approach (Yao *et al*., 2017b). Longer MHs (40 nt versus 20 nt) were shown to be much more efficient in MMEJ‐based knock‐in using human cell lines (Shin *et al*., 2018). However, the same approach performed less efficiently than HR in mouse embryonic stem cells, neuroblastoma and HEK293T cell lines (Yao *et al*., 2017a), indicating either the difference of favourable cell types or growth cycles between HR and MMEJ. MMEJ‐mediated targeted sequence substitutions for therapeutic purposes in genetic disease treatments in vivo would be a great application of the approach. In vivo, MMEJ‐mediated correction of the Fah*^mut/mut^* mouse line, a model of human familial tyrosinemia that produces toxic metabolites leading to fatal consequences, resulted in the survival of the mutant mice (Shin *et al*., 2018). The MMEJ‐based approach was also applied as a biomedical research tool in tracking cells with carnitine acetyltransferase (CAT) knocked out by introducing a fluorescent protein into its ORF using the CRISPR/SaCas9 complex (Katayama *et al*., 2020). Very recently, MMEJ was also shown to be dominant among DSB repaired outcomes of the CRISPR/Cas‐based genome editing in plants (Tan *et al*., 2020; Weiss *et al*., 2020) and could be engineered for precise deletions of plant genomic DNAs (Tan *et al*., 2020) or even chromosomal translocations (Beying *et al*., 2020). MMEJ efficiently involved in repairing DSBs generated by CRISPR/Cas complexes resulted in DNA fragment deletion ranging from some dozens of base pairs to more than 20 kb (Tan *et al*., 2020) or heritable chromosomal translocations in the Mbp range in Arabidopsis (Beying *et al*., 2020). The frequencies of CRISPR/Cas‐based DSB repair by MMEJ were similar to that of cNHEJ and more efficient in the absence of the cNHEJ pathway (Beying *et al*., 2020; Tan *et al*., 2020; Weiss *et al*., 2020).

### A novel system for CRISPR/Cas‐based precision editing via MMEJ

Based on recently published data, we attempt to propose a model for MMEJ‐mediated precision gene insertions or replacements in plants using CRISPR/Cas complexes (Figure 3). One could flexibly set up experiments for targeted insertions (Figure 3a) of specific DNA sequences encoding oligopeptide tags, epitopes or fluorescent protein fusions for tracking the specific location of proteins or targeted replacement of SNPs or alleles (Figure 3b). To do this, a predefined genomic site with a protospacer adjacent motif (PAM) should be first determined; this would enable the DSB formation site to be predicted. The suspected flanking DSB ends would then be used to choose flanking MHs (MH1 and MH2, Figure 3) at different lengths of 8 nt to 20 nt, as suggested by some published data (Nakade *et al*., 2014; Sakuma *et al*., 2016; Yao *et al*., 2017a; Yao *et al*., 2017b). The MHs are then inserted to the two ends of a donor carrying the DNA modifications of interest. The donor is then cloned with synthetic PAM sites added next to the MH terminals and CRISPR/Cas9 gRNA binding sequences that are selected and arranged to perfectly generate the donor DNAs with MHs only inside plant cells. The orientations of Cas9 binding and cutting are designed to avoid recurrent cutting in repaired products (Figure 3).

Some points to be considered for designing experiments for MMEJ‐mediated precision editing in plants: (i) selection of CRISPR/Cas complexes for site‐specific DSB formation, (ii) delivery method for introducing the editing tools into plants, that is via Agrobacterium or particle bombardment‐mediated transformation, or PEG or electroporation‐mediated protoplast transformation, (iii) the type of CRISPR/Cas editing tools to the nucleus and the targeting sites (T‐DNAs, RNPs or DNA replicons), (iv) synchronization or spatial and temporal controls of the expression of Cas proteins, gRNAs and donors, (v) the DNA donor length and (vi) possible methods for quantification MMEJ frequency (NGS, PCR, Southern blot; fluorescence; and GUS assay).

### Strategies to improve CRISPR/Cas‐based precision editing via MMEJ efficiency

It is worth understanding that the MMEJ‐mediated repair mechanism in plants may be different from that of animal systems, as some MMEJ repair components, such as DNA ligase III and SIRT6 proteins, have yet to be identified in plants. Another possibility following from MMEJ is the generation of chromosomal translocation or arrangement, probably due to kinetics of its repair process being slower than the SSA pathway (Sinha *et al*., 2017). Slow kinetics might occur during unfavourable cell cycles (G2) (Figure 2) when MMEJ components are not highly abundant; hence, extensive end resection was observed as evidence of the slow kinetics (Sinha *et al*., 2017).

To overcome these issues, conducting MMEJ‐mediated experiments under favourable conditions would be a key solution. One of the approaches to enhance MMEJ‐mediated DSB repair may be supporting faster DSB end resection by directly providing exonucleases that work on the 3′ end of the DSB terminals. Co‐expressing a human 3′ repair exonuclease 2 (Trex2), with Cas9 protein in *Setaria viridis* resulted in enhancement of targeted mutagenesis frequencies up to 1.7‐folds compared with that of Cas9 alone. More importantly, possibly due to the activity of Trex2, DNA deletions dictated the repaired outcomes at longer deletion lengths, a typical characteristic of MMEJ‐mediated repair of DSBs (Weiss *et al*., 2020). The efficacy and specificity of MMEJ‐mediated repair approaches are highly dependent on the presence of MHs at DSB sites and their characteristics, such as lengths, base composition and distance from the broken ends (Tan *et al*., 2020; Weiss *et al*., 2020). Therefore, precision engineering for the generation of expected modifications at a specific genomic locus may rely on the ability to accurately predict and propose the possibility of MH usage at the locus (Ata *et al*., 2018; Bae *et al*., 2014). Moreover, MMEJ was shown to be preferred in some conditions, such as at specific cell cycles and in certain cell types, genome contexts, donor DNA parameters and cargos as well as with specific mechanisms of delivering editing tools. In addition, blocking the cNHEJ by knocking out the KU70 led to a fivefold enhancement of MMEJ‐based chromosomal translocations in *Arabidopsis* (Beying *et al*., 2020), thus, suggesting an interesting strategy to enhance MMEJ frequency. Harnessing the favourable conditions for MMEJ would further improve its precision editing. It would be useful for plant engineers to determine and optimize the conditions for MMEJ‐based editing since little information has been released about its applications in plants (Beying *et al*., 2020; Tan *et al*., 2020; Weiss *et al*., 2020).

Early data relating to MMEJ showed a preference for sequences from extracellular repaired products of minute virus of mice (MVM) in mouse culture cells (Hogan and Faust, 1984). The MHs TGAC (50% A + T), AATGTTGGTT (70% A + T) and TTTCT (80% A + T) flanking the linear DNA fragment of MVM led to long deletions of intervening sequences at 2740, 2766 and 3395 bp, respectively. The data led to the conclusion that large deletions resulting from MHs at 4‐ to 10‐bp might be A‐T rich (Hogan and Faust, 1984). In human cells, however, GC‐rich MHs located 3 nt from DSB ends were shown to be linked to robust MMEJ repair (Kent *et al*., 2015). MMEJ in yeast, *Drosophila* and mammals was shown to preferentially adopt certain MH patterns (Ata *et al*., 2018; Ma *et al*., 2003; McVey *et al*., 2004). For example, the GC content of microhomologous sequences might determine the repair pathway with a bias for MMEJ (Daley and Wilson, 2005). During the annealing step, the 3′ resected overhangs might be bound by RPA complexes, thereby inhibiting MH annealing. Knockout mutants of RPA showed up to 350‐fold enhancement of MMEJ‐mediated end‐joining frequency (Deng *et al*., 2014). However, RPA complexes are important for DNA replication and other DSB repair pathways, so transient suppression of RPA may be a solution for facilitating MMEJ‐based editing. Highly efficient MMEJ‐mediated precision editing could be carried out by generation of ~20 nt 3′ overhangs with ~5 nt microhomologies in chromosomal acceptors and extrachromosomal DNA donors for avoiding chromosomal rearrangement consequences (Beall and Rio, 1997; McVey and Lee, 2008).

Mapping of the MHs among DSB repair outcomes by deep sequencing techniques has been very powerful for understanding their distributions and selections for annealing during MMEJ. Analysis of CRISPR/Cas9‐based DSB repair outcomes by NGS revealed and validated the major involvement of MMEJ in the repair process and generated corresponding maps of MH usage in human cells (Bae *et al*., 2014) and plants (Tan *et al*., 2020; Weiss *et al*., 2020). An online bioinformatics tool for predicting MHs and scoring has also been established based on the collected data. However, the MH prediction and sorting criteria of Bae and co‐workers might not be sufficient for high confidence prediction of MMEJ assay results, since at least 4000 microhomology scored MHs did not facilitate dominant MMEJ outcomes in zebrafish embryos (Ata *et al*., 2018). Ata and co‐workers improved Bae’s protocol for MH scoring to predict MMEJ‐mediated precision editing in zebrafish. In particular, the higher the number of local MHs with similar abundances was, the lower the MMEJ activation frequencies were. In contrast, MMEJ activation was higher with fewer competing MHs. Essentially, only one or two MH pairs presented at DSBs would strongly activate MMEJ. In addition, the distance between MHs flanking DSBs was important for the prediction of MHs involved in MMEJ, since only MHs with less than 5 bp intervals could be precisely predicted for their involvement in the MMEJ process. Likewise, a program was developed for the prediction of MMEJ‐mediated CRISPR/Cas‐based editing with specific targeted sequences at high accuracy (Ata *et al*., 2018). It seems that intrachromosomal MMEJ was preferentially activated by short MHs (2–8 nt) (Ata *et al*., 2018; Bae *et al*., 2014), whereas in intermolecular gene knock‐in approaches (i.e. donor DNAs and genomic loci) longer MHs might be required, as they showed better performance that was proportional to donor lengths (Sakuma *et al*., 2016; Yao *et al*., 2017a).

Other MMEJ improvement approaches might be connected to early or late steps of the repair process. K48‐linked polyubiquitylation‐mediated removal of KU80 from DSB ends may promote alternative repair pathways (Postow *et al*., 2008). Suppression of KU80 accumulation at DSB sites prevents it from forming a heterodimer with KU70, thereby supporting end resection for MMEJ and HR. Calicheamicin gamma 1 was reported to stimulate the (poly)ADP‐ribosylation activities of PARP‐1, which could strongly compete with other DSB sensors, such as the KU complex, for supporting MMEJ repair. Facilitating the gap‐filling step by overexpression of *S. cerevisiae* POL3/CDC2 (Galli *et al*., 2015; Giot *et al*., 1997) may also enhance precision repair by competing with translesion DNA polymerases such as Pol θ (Kent *et al*., 2015; Newman *et al*., 2015).

## Concluding remarks and future perspectives

MMEJ‐based gene editing offers alternative tools for precision engineering of organisms of interest at a potentially higher frequency than what is achieved with HR‐based approaches. Substantial understanding of MMEJ activation and its mechanism in DSB repair (Figure 1) has been made in animal and plant studies (Ata *et al*., 2018; Bae *et al*., 2014; Beying *et al*., 2020; Sfeir and Symington, 2015; Tan *et al*., 2020; Weiss *et al*., 2020). Therefore, subsequent applications in precision gene knock‐in using animals showed significantly higher efficiencies than that of HR in the same experimental conditions. Nevertheless, experimental parameters that affect MMEJ‐mediated DSB repair were shown to vary according to different MH length, composition, and distribution, donor size, experimental condition and cell/tissue/animal model. From our point of view, MMEJ‐mediated repair could be well adapted to CRISPR/Cas‐based precision plant genome editing techniques as an alternative to HR. However, due to the lack of published data on MMEJ applications in plants, one should begin such tests in plants with extensive optimization according to the abovementioned parameters. There is still room for improvement of engineering MMEJ in plants, as its mechanism and molecular components and signalling are increasingly revealed. Because HR‐mediated editing in plants is still a challenge, the MMEJ approach may extend options for precision plant genome engineering.

Since the advent of CRISPR/Cas technology, DSB formation has become much more precise, flexible and customizable. In general, both blunt‐end and cohesive‐end DSBs can be used in MMEJ‐mediated editing, but the former type might be easier to design and might produce more predictable repair outcomes. Moreover, due to the need for end resection to generate 3′ overhangs, Cas complexes that produce 5′ overhangs, such as Cas12a, may not be energetically preferred. The DSB inducers and donor DNAs are conventionally introduced into plants by *Agrobacterium*‐mediated transformation or particle bombardment or protoplast transfection; *Agrobacterium*‐mediated transformation is the most widely used thanks to its ease and low cost. However, *Agrobacterium*‐mediated transformation usually delivers low copy numbers of the editing tools in its T‐DNA system, thereby limiting the competitiveness of exogenous donors to prevent re‐ligation of a broken end by cNHEJ. Recent advances in HR works have used DNA replicons as efficient donor DNA cargo for plant genome editing (Baltes *et al*., 2014; Cermak *et al*., 2015; Vu *et al*., 2020). The DNA replicons could amplify the donor DNA to hundreds or thousands of copies per cell, which were readily able to function in the repair process. Furthermore, the localization of donor DNAs to targeted sites and synchronization of DSB formations of the donor DNA cargos and genomic loci should be considered advancements of the MMEJ‐mediated genome engineering approach.

In this review, we summarize the MMEJ‐mediated DSB repair mechanism at each step that requires the involvement of a cascade of DNA damage repair proteins/enzymes (Figure 1). MMEJ‐mediated repair requires as few as one base pair MHs for amending a DNA DSB, but a substantial MH length is highly preferred for efficient and hence predictable repairs (Ata *et al*., 2018; Bae *et al*., 2014). MH lengths have been characterized at longer sequences for efficient precision gene knock‐in in animals, which might be one of the essential requirements for the insertion of long donor DNAs into DSB sites via MMEJ (Nakade *et al*., 2014; Sakuma *et al*., 2016; Shin *et al*., 2018; Yao *et al*., 2017b). The recent progress of precision genome editing via the MMEJ pathway is therefore updated. From the validated data, we attempted to generalize a system design for MMEJ‐mediated CRISPR/SpCas9‐based precision insertion and replacement (Figure 3), since the DSB sites for the introduction of DNA donor cargo and genomic loci must be smartly arranged to avoid recurrent cutting after repair (Nakade *et al*., 2014). However, because the published data on MMEJ applications in plants is limited (Beying *et al*., 2020; Tan *et al*., 2020; Weiss *et al*., 2020), one should begin with extensive optimization according to the abovementioned parameters. The system could be readily applied for precision plant genome editing as an alternative to the HR editing approach. Future perspectives and improvement of the system offer a promising novel tool for precision plant breeding.

## Conflicts of interest

No conflicts of interest are declared.

## Author contributions

Conceptualization, T.V. V and J.Y.K.; Methodology, T.V. V and J.Y.K.; Writing—Original Draft, T.V. V., D.T.H. D., J.K., Y.W.S., M.T.T., Y.J.S. and S.D.; Writing—Review and Editing, T.V. V., D.T.H. D., J.K., S.D. and J.Y.K.; Funding Acquisition, J.Y.K.; and Supervision, T.V. V and J.Y.K.
